# Subgenome evolution in allotetraploid plants

**DOI:** 10.1111/tpj.15190

**Published:** 2021-03-24

**Authors:** Matteo Schiavinato, Alexandrina Bodrug‐Schepers, Juliane C. Dohm, Heinz Himmelbauer

**Affiliations:** ^1^ Department of Biotechnology University of Natural Resources and Life Sciences (BOKU) Institute of Computational Biology Muthgasse 18 Vienna 1190 Austria

**Keywords:** interspecific hybrid, allotetraploid, genome evolution, subgenome intermixing, domestication, crop plant

## Abstract

Polyploidization is a well‐known speciation and adaptation mechanism. Traces of former polyploidization events were discovered within many genomes, and especially in plants. Allopolyploidization by interspecific hybridization between two species is common. Among hybrid plants, many are domesticated species of agricultural interest and many of their genomes and of their presumptive parents have been sequenced. Hybrid genomes remain challenging to analyse because of the presence of multiple subgenomes. The genomes of hybrids often undergo rearrangement and degradation over time. Based on 10 hybrid plant genomes from six different genera, with hybridization dating from 10,000 to 5 million years ago, we assessed subgenome degradation, subgenomic intermixing and biased subgenome fractionation. The restructuring of hybrid genomes does not proceed proportionally with the age of the hybrid. The oldest hybrids in our data set display completely different fates: whereas the subgenomes of the tobacco plant *Nicotiana benthamiana* are in an advanced stage of degradation, the subgenomes of quinoa (*Chenopodium quinoa*) are exceptionally well conserved by structure and sequence. We observed statistically significant biased subgenome fractionation in seven out of 10 hybrids, which had different ages and subgenomic intermixing levels. Hence, we conclude that no correlation exists between biased fractionation and subgenome intermixing. Lastly, domestication may encourage or hinder subgenome intermixing, depending on the evolutionary context. In summary, comparative analysis of hybrid genomes and their presumptive parents allowed us to determine commonalities and differences between their evolutionary fates. In order to facilitate the future analysis of further hybrid genomes, we automated the analysis steps within manticore, which is publicly available at https://github.com/MatteoSchiavinato/manticore.git.

## Significance Statement

The purpose of this work was to compare the level of subgenomic intermixing in hybrid plants, providing insight into how intermixing levels are influenced by domestication, biased fractionation or hybrid age. A computational framework is presented to evaluate subgenomic conservation and intermixing in other hybrids, as there seems to be no one rule that fits all.

## INTRODUCTION

Polyploidization is a common speciation mechanism in plants that is considered to account for about 50% of the speciation events in angiosperms (Soltis *et al.*, 2015). Polyploidization may reproductively isolate a newly formed hybrid, leading to the generation of a new species if the hybrid is not sterile and reproduces with itself (self‐fertilization) or with other hybrids of the same type. The two main sources of polyploidization are whole‐genome duplication (WGD), resulting in autopolyploidization, and hybridization between different species, leading to allopolyploidization (Thompson and Lumaret, 1992). Both create an excess of genetic material by increasing the ploidy of the genome and have often been associated with speciation or adaptation events (Becker *et al.*, 2013; Soltis *et al.*, 2015). This is a challenge for a cell undergoing cell division, as chromosome pairing is hindered by the presence of more than two copies of the same chromosome (i.e. the homeologs), and this may lead to disadvantages or lethal rearrangements. A tetraploid genome tends to diploidize over time, reinstating the normal ploidy of the cell. In fact many species, including the ancestor of all vertebrates, are believed to have undergone rounds of whole‐genome duplication, although their genomes are now diploid (Dehal and Boore, 2005). The newly acquired genetic material may also be advantageous, however (Cheng *et al.*, 2018; Panchy *et al.*, 2016). WGD has contributed to the survival of many plant species, whereas hybridization has been at the root of many speciation events (Soltis and Soltis, 2009). For example, the autotetraploid Rangpur lime (*Citrus limonia*) has better drought resistance than its diploid relative (Allario *et al.*, 2013), and the allopolyploidization of white clover (*Trifolium repens*) has been recently shown to have facilitated its expansion into new ecological niches (Griffiths *et al.*, 2019). Despite numerous studies on polyploid species, the dynamics that influence the genome architecture and sequence conservation of hybrid genomes during their evolution have yet to be fully understood.

The most distinctive trait of hybrid species is the coexistence of multiple ‘parental genomes’ within the same nucleus, referred to as subgenomes. Each subgenome has had an independent evolution before having become united in the hybrid (Comai, 2005). The sudden change in ploidy triggers a ‘genomic shock’ (McClintock, 1984) that leads to unpredictable genome rearrangements, some of which may already take place a few cell divisions after the hybridization occurred (Jackson and Chen, 2010; Wendel *et al.*, 2016). Hence, there may be substantial loss of synteny between a parental progenitor’s genome and the corresponding subgenome in the hybrid. On the other hand, the interfertility between progenitors suggests their close phylogenetic relationship, which in turn translates into high sequence identity between their genomes.

The loss of synteny, together with the high identity between subgenomes, poses a great challenge when analysing the architecture of hybrid genomes. The loss of synteny limits structure comparisons against the parental genomes, which are based on long stretches of conserved DNA sequence. The high identity between subgenomes may lead to fusion sequences between the subgenomes, instead of assembling homoeologous chromosomes separately. The International Wheat Genome Sequencing Consortium faced an immense task during the analysis of the hexaploid wheat (*Triticum aestivum*) genome, which took many years (The International Wheat Genome Sequencing Consortium (IWGSC) *et al.*, 2018). Others chose to wait for the availability of more advanced sequencing tools before trying to assemble a polyploid genome of interest. For instance, sequencing the recently completed genome of the tetraploid domesticated peanut (*Arachis hypogaea*) (Bertioli *et al.*, 2019) had been on hold for several years, as it became clear that it would not be easy with the technology available at the time. Therefore, the wild diploid parents of cultivated peanut were sequenced first (Bertioli *et al.*, 2016).

In order to study individual subgenomic properties, the subgenomes need to be distinguished from each other. A series of *in silico* methods have been developed to attempt the separation of subgenomes of hybrid species. Some methods require genomic sequencing data from the parental progenitor species (Page *et al.*, 2013) or gene sets and assemblies when the separation is based on synteny (Bertioli *et al.*, 2019; Edger *et al.*, 2019; Gordon *et al.*, 2020). Others require variant calling from genomic or transcriptomic sequencing reads (Khan *et al.*, 2016; Peralta *et al.*, 2013), or the construction of a set of phylogenetic trees (Schiavinato *et al.*, 2020).

Regardless of the method of choice or the age of the hybrid, a non‐negligible fraction of the hybrid genome often remains unassigned to one of the parents (Edwards *et al.*, 2017; Jarvis *et al.*, 2017; Schiavinato *et al.*, 2020). Some of these methods require substantial data preparation before their usage, be it gene annotation, the construction of phylogenetic trees or genome‐wide variant calling. Other methods, such as those based on raw sequencing reads from the parents, use variant calling algorithms to separate raw reads into parental read pools, which must be analysed by the user later on. Hence, even in settings with well‐documented parental lineages, an absence of backcrossing or introgression and a known hybridization date, studying the subgenomes of a hybrid remains challenging.

One of the factors influencing subgenome separation is the extent of genetic material exchange that took place between them since the generation of the hybrid, a phenomenon referred to as ‘subgenomic intermixing’ or ‘homoeologous exchange’, which is the consequence of recombination between homoeologous chromosomes (Mason and Wendel, 2020). One of the most studied genera to understand the effects of polyploidization is the genus *Nicotiana* (Kelly *et al.*, 2013). More than a decade ago, Lim *et al.* (2007) showed that the genomes of allotetraploid species in *Nicotiana* section *Repandae* have exhibited a remarkably high rate of subgenomic intermixing since its emergence 5 million years ago. In previous work we have shown that this is also the case for *Nicotiana* section *Suaveolentes*, which is of similar age (Schiavinato *et al.*, 2020). In *Nicotiana benthamiana*, a species affiliated with this section, subgenome identification based on parental read mapping failed, but succeeded with a phylogenomic approach, i.e. through the analysis of a genome‐wide collection of phylogenetic gene trees (Schiavinato *et al.*, 2020). However, in the similarly aged allotetraploid *Chenopodium quinoa*, for which the hybridization event has been estimated as 3.3–6.3 million years ago (Mya), the separation of subgenomes based on sequence identity to the parental genomes was successful (Jarvis *et al.*, 2017). This difference shows the importance of analysing the intermixing state of the subgenomes within a hybrid. Several factors, such as sequence similarity between the parental genomes and genomic loss following hybridization, influence how well subgenomes can be distinguished and, as a consequence, how successfully the intermixing state can be studied. Here, we try to uncouple these factors to better isolate and observe the intermixing effect using a custom read‐mapping approach that requires the genome sequence of a hybrid and genomic sequencing reads from its candidate parental progenitors. The architecture and conservation of the subgenomes of the hybrid are analysed without the need to address genome or gene‐based synteny, thereby reducing the number of steps and automating the analysis from the raw data to the results. We discuss the results in light of known evolutionary mechanisms such as domestication, biased fractionation, self‐fertilization and niche adaptation. We include allopolyploid plant species for which a genome assembly and short sequencing reads of the extant descendants of the parental progenitors are available and the age of hybridization is known, that is, *Arachis hypogaea* (cultivated peanut), *Arachis monticola* (wild peanut), *Brassica juncea* (Chinese mustard), *Brassica napus* (rapeseed), *Chenopodium quinoa* (quinoa), *Gossypium hirsutum* (upland cotton), *Nicotiana benthamiana* (native Australian tobacco), *Nicotiana tabacum* (smoking tobacco), *Triticum turgidum* ssp. *dicoccoides* (wild emmer wheat) and *Triticum turgidum* ssp. *durum* (domesticated emmer wheat) (Table S1). By mapping the parental genomic sequencing reads onto the genome assembly of the hybrid we generate coverage profiles for the subgenomes of these species. We compute several coverage‐based metrics to evaluate the individual subgenomic conservation and the subgenomic intermixing state in each of the analysed hybrids. Our analysis pipeline is compiled in a program called manticore that we provide with this article.

## RESULTS

### Case study: hybrids from the *Brassica* genus

The well‐studied genus *Brassica* encompasses several diploid species, some of which have been involved in hybridization events as parental donors. Three relevant species are *Brassica* *oleracea* (cabbage; subgenome A donor), *Brassica* *nigra* (black mustard; subgenome B donor) and *Brassica* *rapa* (field mustard; subgenome C donor). Their hybrids are *Brassica* *napus* (rapeseed; AC hybrid), *Brassica* *juncea* (Chinese mustard; AB hybrid) and *Brassica* *carinata* (Abyssinian mustard; BC hybrid). The genomes of rapeseed and Chinese mustard have been sequenced and their hybridization age is known (Kim *et al.*, 2018; Nagaharu, 1935).

We took advantage of the availability of short‐read data, assemblies and hybridization information on rapeseed, Chinese mustard and their respective parental subgenome donors to analyse subgenomic sequence conservation, structure conservation and subgenomic intermixing, based on read coverage from the parental species. In our approach, we map the parental reads against the hybrid genome sequence and analyse a series of metrics in intervals of 250 Kbp (interval size set as input parameter) along the hybrid genome to study the subgenomes. For each interval we compute the number of positions covered by reads from either parental read data set and determine the fraction of the total positions covered by those reads, referred to as ‘covered fraction’ for each parent independently. Additionally, the mean coverage per position in each interval is calculated considering covered positions only. This second metric allows us to identify intervals that have an observed coverage close to the expected coverage. Mean coverage and covered fraction combined highlight intervals that are more likely to have derived from one parent than from the other: in such intervals one parent would show an observed coverage closer to the expected coverage and a higher fraction of covered positions, in contrast to the other parent.

In some cases, two parental progenitor read sets can both contribute to the coverage of an interval if the genome rearrangements between subgenomes have taken place within that region of the hybrid genome. Such situations are not uncommon in complex plant genomes and are at the core of this study. It is within those intervals that we observe what we refer to as ‘subgenomic intermixing’, i.e. a contribution from both parental progenitors to a certain genomic region in the hybrid. It could happen that adjacent portions of the same chromosome represent well‐conserved regions from different subgenomes brought together by a homoeologous recombination event (i.e. intermixing). As a result of the sequence identity between homeologous chromosomes, it is likely that these intermixed regions will be covered by the sequencing reads of both parents. Nevertheless, for any region of hybrid DNA we can also expect that genomic reads from the correct parent of the region will, in most cases, map with fewer mismatches or gaps onto these regions, producing a higher mean coverage.

**Figure 1:**
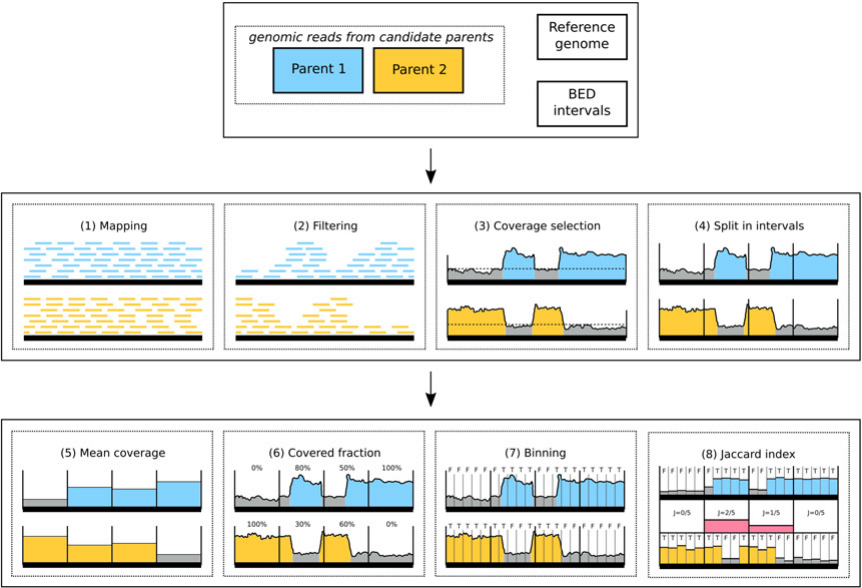
Analysis steps implemented in the manticore pipeline. Top panel: input files include short genomic sequencing reads from two parental progenitor species, a reference genome assembly in FASTA format for the hybrid and genome coordinates to consider in the analysis (optional) in BED format. Middle panel: 1–4, main data‐processing steps. The horizontal bold line represents the hybrid genome sequence; reads of the two parental genomes are mapped to the hybrid independently. The horizontal dashed black line in panel 3 represents the coverage threshold below which data is not considered. The vertical lines in panel 4 represent interval boundaries. Bottom panel: 5–8, metrics computed by manticore. Additional vertical lines in panels 7‐8 represent subdivisions into bins for Jaccard index calculation along the four genomic intervals. Blue and yellow: coverage profiles obtained from alignment of sequencing data from the two parents. Red: Jaccard index (represented by the height of the red block) in the four intervals. High Jaccard indices indicate a high level of intermixing, i.e. both parents share covered regions that are not necessarily overlapping in the genomic interval.

To quantify subgenomic intermixing we divide each interval into smaller bins and determine the fraction of bins that contain coverage from both parents. Intermixed regions contain stretches of DNA covered by one parent interleaved with stretches covered by the other parent. Such a transition from one to the other parent can be detected as a bin where both parents contribute to the coverage (Figure 1). Thus, if several bins within an interval contain a coverage switch, the interval is considered to be more intermixed than intervals with fewer or no bins containing coverage switches. The fraction of such bins is calculated as the Jaccard index: the number of bins with coverage from both parents divided by the number of bins with coverage from at least one parent. A Jaccard index value close to 0 represents low intermixing, whereas a value close to 1 represents high intermixing. These values can be interpreted as percentages describing the fraction of an interval that shows an intermixing signal. The distributions of mean coverage, covered fraction and Jaccard index from all intervals are used to draw conclusions on the subgenomic intermixing state of the hybrid genome.

**Figure 2:**
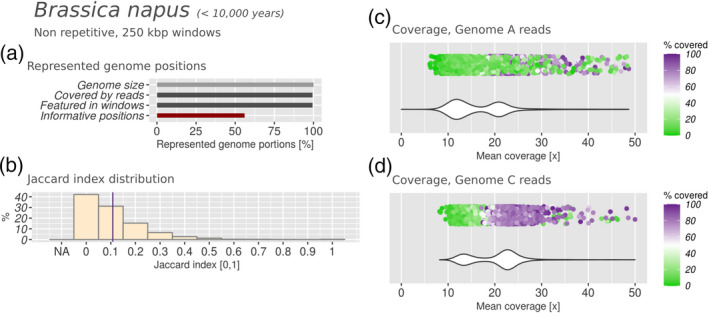
Subgenomic intermixing metrics for rapeseed (*Brassica napus*). Hybrid age is indicated next to the name of the taxon. (a) Represented genome portions. (b) Jaccard index distribution. Purple line: mean Jaccard index. NA: windows with insufficient coverage to compute a Jaccard index. 0: no intermixing. 1: highly intermixed. (c, d) Coverage distribution obtained by mapping of Illumina reads from candidate parental species against the hybrid genome assembly. The dot plot on top represents the covered fraction in each interval of 250 kbp (with one dot per interval). Colour range: from green (uncovered) to purple (completely covered). The violin plot below represents the density of intervals at any given coverage.

We mapped genomic short reads from *B. oleracea* (A) and *B. rapa* (C) against the assembly of rapeseed (AC hybrid) and determined the sequencing read coverage (Figure 2). We applied the same approach on Chinese mustard (AB hybrid) using reads from its parental progenitors *B. oleracea* (A) and *B. nigra* (B) (Figure S1). For both hybrids we analysed the read coverage of the parental data sets, in intervals of 250 kbp along the assembled genomes, for the mean coverage and the fraction covered in each interval. When addressing sequence conservation, the expectation is that highly conserved subgenome sequences would result in a bimodal distribution of mean coverage and covered fraction from one parental data set across all intervals: the reads will map successfully to the corresponding conserved subgenomic sequences (peak at a higher coverage) but leave the other subgenome poorly covered (additional peak at a lower coverage). The distribution shifts towards unimodal (single peak at low coverage) when the subgenomic sequences are not well conserved. One should keep in mind that high sequence conservation within intervals of 250 kbp may not imply high structure conservation. In fact, intervals originally deriving from different subgenomes may now be interleaved within the same hybrid chromosome through chromosomal rearrangements, without accumulating point mutations. We observed a bimodal mean coverage and covered fraction distribution for both parental read data sets of *B. napus* (Figure 2c,d), indicating substantial sequence conservation between the subgenomes and their parental counterparts. In Chinese mustard, subgenome B reads resulted in a clear bimodal distribution of mean coverage and covered fraction; the same was true for subgenome A reads, although less sharply (Figure S1c,d).

The Jaccard index distribution indicated that most intervals in rapeseed had ≤15% intermixing signal (mean approx. 0.108; Figure 2b). In other words, most 250‐Kbp intervals were covered by a single parental read data set for ≥85% of their length. These results were obtained from the non‐repetitive positions annotated in the *B. napus* genome assembly, accounting for approximately 55% of the sequence, and distributed across the genome in a way that allowed us to process the entire assembly (Figure 2a, ‘Informative positions’ versus ‘Featured in windows’). Interestingly, the Jaccard index distribution obtained in Chinese mustard (mean approx. 0.04) indicated a lower rate of subgenome intermixing in *B. juncea* than in *B. napus* (Figure S1b). Altogether, these results indicated that there is still high identity between the subgenomes of these two species and their parental genomes, both in terms of sequence conservation as well as in terms of genome structure.

**Figure 3:**
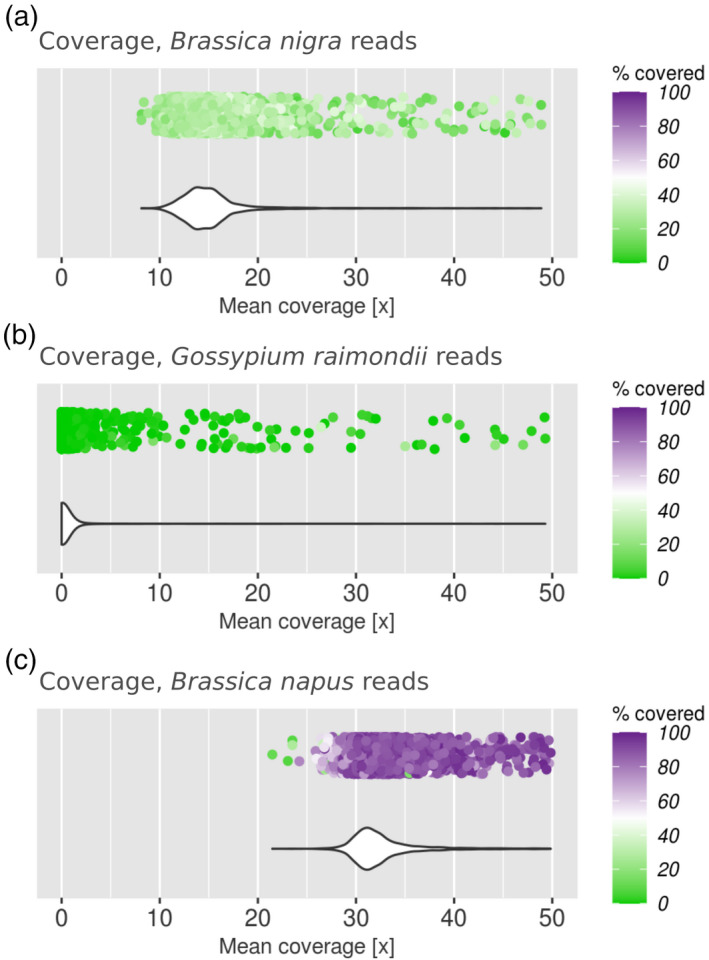
Coverage profile analysis performed on the genome assembly of allotetraploid rapeseed (*Brassica napus*, AC hybrid) using different sets of genomic reads not derived from its parents (subgenomes A or C, respectively; Figure 2): (a) closely related diploid species *Brassica nigra* (subgenome B); (b) unrelated species of upland cotton (*Gossypium* *hirsutum*); and (c) rapeseed. No data set (a–c) permits distinguishing the two rapeseed subgenomes A and C from each other. Details as described in Figure 2.

We then mapped genomic reads from species other than the progenitor genomes against the rapeseed assembly. The species were: (i) distantly related (*Gossypium raimondii*, affiliated with the order Malvales rather than Brassicales); (ii) equally related to both parental genomes (*B. nigra*, subgenome B of Chinese mustard); and (iii) isogenic to the reference genome (i.e. *B. napus* sequencing reads used to calculate the assembly). As expected, none of these three read data sets resulted in a bimodal distribution of mean coverage and covered fraction, and failed to detect the two subgenomes within the *B. napus* genome (Figure 3). We concluded that only the correct parental progenitors, or very closely related candidate species, could result in a bimodal coverage distribution when mapped onto the genome assembly of a hybrid.

A strength of the method presented here is that it does not need gene models or assemblies to infer the parental origin of a hybrid genome region, while previous studies largely used them for this purpose. To compare the result of our read mapping‐based approach to subgenome separation based on genes, we downloaded the sequences of 101 040 *B. napus* gene models (Chalhoub *et al.*, 2014). We used the assigned intervals of the genome of *B. napus* obtained with our manticore pipeline to assign a parental origin to the genes contained in each interval. We only considered intervals where intermixing was observed for less than 50% of their length (i.e. a Jaccard index of <0.5) and where more than half of the interval was covered by reads from one parent (827 Mb, 97% of the *B. napus* genome assembly). Based on this, we assigned 42 870 genes to subgenome A and 55 043 genes to subgenome C (97 913 in total of 101 040 genes, 96%). There was no clear assignment for the remaining genes for two reasons: 961 genes extended across a border between two intervals that had been assigned to different parents, whereas 2166 genes could not be assigned because the corresponding interval was not assigned either. We then compared the transcript sequences of *B. napus* with the transcripts of its parental progenitors. Based on sequence homology (see Experimental procedures) we assigned 43 226 genes to subgenome A and 53 493 genes to subgenome C (96 719 genes in total, 95%). There was no assignment possible if no matching sequence was found in either parent (3582 *B. napus* transcripts) or if both parental copies were equally similar to the same *B. napus* gene (745 *B. napus* transcripts). The two methods could be compared for a total of 93 760 gene models that had an assignment with both methods, and 81 570 of these (87%) were in agreement.

The 12 362 gene models that were differently assigned by the two methods were further inspected. We compared the subgenomic assignment of such genes with the subgenomic assignment of the chromosome containing them according to the chromosome name in the genome assembly. For nearly all of these genes (98%) the assignment to a certain subgenome by manticore corresponded to the subgenomic assignment of the whole chromosome, whereas the assignment with gene models corresponded to the other subgenome. Hence, this indicated a different subgenomic origin for these genes relative to the surrounding sequence that they are embedded in. These genes may simply flag instances where assembly errors have occurred. However, a more intriguing explanation would be that these genes may have been subjected to gene conversion. We found the mean Jaccard index to be slightly higher (0.12) in the intervals where these discordantly assigned genes were located, compared with intervals where assignments were in agreement in both methods (0.10). Decreasing the interval size to 25 Kbp with the read‐mapping approach yielded similar results, showing that this is not an artifact produced by the large 250‐Kbp intervals. All in all, from these results we concluded that our analysis strategy based on read mapping was appropriate to determine the subgenome composition of hybrid genomes in terms of coverage and intermixing state, without the need to consider gene models.

We note that our approach and the gene‐based approach for subgenome assignment have different qualities and pitfalls. The assignment of genes by homology is a very sensitive approach that can be influenced by differences as small as one single mutation. This powerful strategy is prone to errors in the presence of homoeologs because genes in a subgenome may be converted to the homoeologous version, by a mechanism called gene conversion (Flagel *et al.*, 2012; Wendel, 2000). The assignment of genomic intervals based on our read‐mapping approach is robust against the impact of single gene conversion events but does not allow their detection on its own. Hence, the two strategies can complement each other in cases where gene models have been annotated in the genome of a hybrid, and information on the parental origin of these genes exist.

### Subgenomic intermixing and hybrid age

Two polyploid species with similar hybridization age might display different levels of subgenomic intermixing, as exemplified by the lower intermixing level observed in Chinese mustard when compared with its relative rapeseed. To further explore the relationship between hybridization age and intermixing, we applied our method on two similarly aged allotetraploid hybrids: the Australian tobacco species *N. benthamiana* (hybridization dated 5 Mya) and the well‐known South American pseudocereal quinoa (*C. quinoa*, 3.3–6.3 Mya) (Jarvis *et al.*, 2017; Schiavinato *et al.*, 2020). Contrary to the *Brassica* species described above, these two species belong to distantly related genera, the most recent common ancestor of which is the ancestor of the Pentapetalae clade, from which they separated 121–126 Mya (Magallón *et al.*, 2015). The two diploid parental progenitors of *N. benthamiana* are affiliated with the *Nicotiana* sections *Noctiflorae* and *Sylvestres*, respectively (Schiavinato *et al.*, 2020). Previous studies have highlighted how the *Nicotiana* genus shows high genome plasticity (Bally *et al.*, 2015; Leitch *et al.*, 2008; Lim *et al.*, 2007). In fact, the separation of the *N. benthamiana* subgenomes by parental read mapping was unsuccessful because of excessive divergence between the evolved subgenomes and their corresponding parental donors (Schiavinato *et al.*, 2020). On the contrary, the separation of the quinoa subgenomes was successfully achieved (Jarvis *et al.*, 2017). Although the original parental species that hybridized during the formation of allotetraploid quinoa are unknown, extant related species are available. *Chenopodium pallidicaule* is a close relative of the quinoa subgenome A donor, whereas the subgenome B parent has its match with *Chenopodium ficifolium* or *Chenopodium suecicum* (Walsh *et al.*, 2015). Genomic sequencing reads from *C. suecicum*, together with sequencing data from *C. pallidicaule*, were used to separate subgenomes A and B of quinoa (Jarvis *et al.*, 2017).

**Figure 4:**
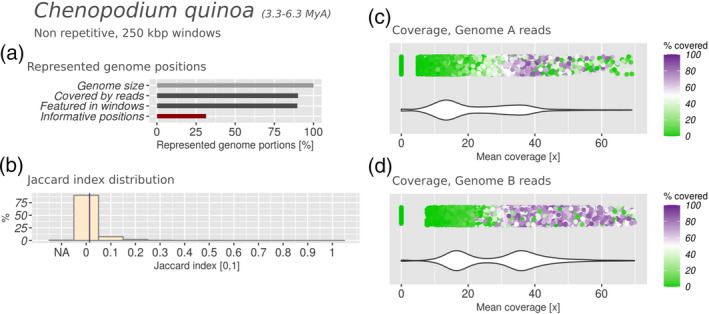
Subgenomic intermixing metrics for quinoa (*Chenopodium quinoa*). Details as described in Figure 2.

**Figure 5:**
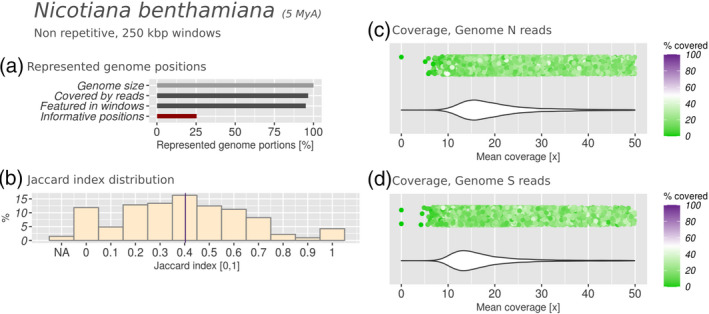
Subgenomic intermixing metrics for native Australian tobacco (*Nicotiana benthamiana*). Details as described in Figure 2.

We analysed the genomes of both *N. benthamiana* and *C. quinoa*, assessing parental read coverages in intervals of 250 Kbp along the reference genome assemblies of these hybrids. For *N. benthamiana* we used genomic reads from two extant relatives of the parental genomes, i.e. *Nicotiana glauca* (section *Noctiflorae*) and *Nicotiana sylvestris* (section *Sylvestres*), respectively. For quinoa we used genomic reads from *C. pallidicaule* and *C. suecicum* (Figure 4). Our results show that in both hybrid species, 25–30% of the genomic positions were covered by reads (Figures 4 and 5). These positions were distributed evenly over the genome, allowing us to process 85–90% of the total genome in intervals of 250 Kbp with sufficient read coverage (Figures 4a and 5a). The Jaccard index distribution for *N. benthamiana* had a mean of 0.403, the highest such value registered in our study, indicating a high level of subgenome intermixing (Figure 5b). Additionally, the unimodal and broad distributions of mean coverage and covered fraction from both parental read data sets indicated high diversification on the sequence level (Figure 5c,d). Given the confidence we have in the parental lineage (Clarkson *et al.*, 2017; Goodin *et al.*, 2008; Schiavinato *et al.*, 2020), this is likely to be a result of the rapid accumulation of genomic mutations and rearrangements in the subgenomes compared with their parental progenitors. The rather unimodal and broad distribution of the covered fraction suggests that there is no part of the hybrid genome sequence that clearly resembles the parental progenitors anymore. This is also true when narrowing down the analysis to coding regions (CDS), which are more conserved than non‐coding regions (Figure S6). The unimodal distribution of the mean coverage instead suggests uniform shift from the parental sequence and structure, hindering the distinction between genomic regions derived from different parents (Figure 5).

In quinoa, on the contrary, the mean Jaccard index was close to zero (0.015, Figure 4b) and represented the lowest level of intermixing detected in our study. The parental read coverage distributions are bimodal, reflecting subgenomes that are well conserved in sequence and easily identifiable with parental sequencing reads (Figure 4c,d). Notably, the peaks obtained with subgenome B reads were sharper than those obtained with subgenome A reads. In subgenome A the mode corresponding to the highly covered intervals (approx. 35×) is almost flattened, whereas the mode corresponding to poorly covered intervals (approx. 15×) is still present. This could indicate the higher loss or remodelling of the quinoa subgenome A, but also a suboptimal choice of the parental progenitor candidate (Heitkam *et al.*, 2020).

As a means of comparison, we analysed the subgenomes of two other species: first, the smoking tobacco *N. tabacum*, a young hybrid from the *Nicotiana* genus with its hybridization event dated to 0.4 Mya (Clarkson *et al.*, 2017; Schiavinato *et al.*, 2020); and second, the upland cotton *G. hirsutum*, with hybrid formation dated to 1.5 Mya (Li *et al.*, 2015). To analyse the genome of smoking tobacco we used reads from its parents *N. sylvestris* and *Nicotiana tomentosiformis* (Sierro *et al.*, 2013). The trends observed from this species resemble those observed in quinoa: the Jaccard index distribution showed little intermixing, and one of the two subgenomes (subgenome S) is more degraded than the other (Figure S2c,d). We note that the fraction of genome positions covered by reads (60–65%) was much larger than the fraction obtained with quinoa or *N. benthamiana*. However, the distribution of positions featured in 250‐Kbp intervals was comparable (85–90%; Figure S2a).

To analyse the genome of upland cotton we used reads from its parents *Gossypium arboreum* and *G. raimondii*. As for quinoa, the results provide evidence for remarkably well‐separated subgenomes (Figure S3), indicating that the subgenomes of quinoa and upland cotton have been kept apart in their non‐repetitive portions, and that subgenome separation was successful with both parental sequencing reads. Taken together, the results observed in quinoa, upland cotton and smoking tobacco are comparable, despite the large differences in hybridization age. These results diverged sensibly from the result for *N. benthamiana*, even with the similarly aged quinoa. We concluded that time alone cannot explain the level of observed subgenomic intermixing in a hybrid species, especially when looking at species belonging to different genera.

### Intermixing and domestication

Many domesticated crops have polyploid genomes. Salman‐Minkov *et al.* (2016) examined polyploidy in genera containing at least one major crop species and found polyploids more often among domesticated species than among their wild relatives. Hence, we set out to identify whether any interplay exists between domestication and the conservation of subgenomic sequence and structure. We applied our method to two well‐characterized cases where polyploid domesticated species, their wild relatives and the parental species have been sequenced and were publicly available: peanut (genus *Arachis*) and emmer wheat (subspecies of *T. turgidum*).

In the genus *Arachis*, the wild peanut *A. monticola* is considered to be the direct ancestor of the domesticated peanut *A. hypogaea* (Yin *et al.*, 2018). Wild peanut is an allotetraploid species that arose by hybridization between ancestors of the extant species *Arachis duranensis* (subgenome A) and *Arachis ipaensis* (subgenome B); this hybridization event has been dated to 10 000 years ago, followed shortly by domestication, giving rise to the cultivated peanut (Bertioli *et al.*, 2019). What happens to allotetraploid genomes in the wild, as opposed to 10 000 years of cultivation?

**Figure 6:**
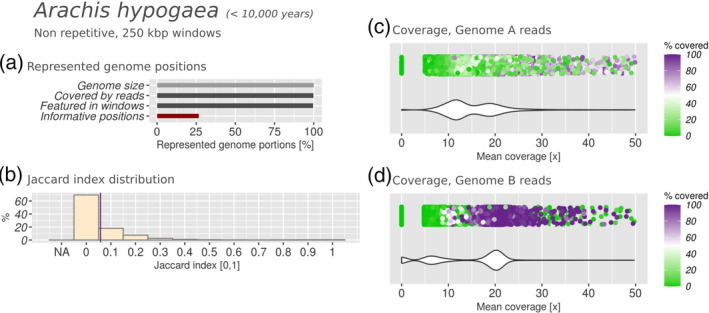
Subgenomic intermixing metrics for domesticated peanut (*Arachis hypogaea*). Details as described in Figure 2.

**Figure 7:**
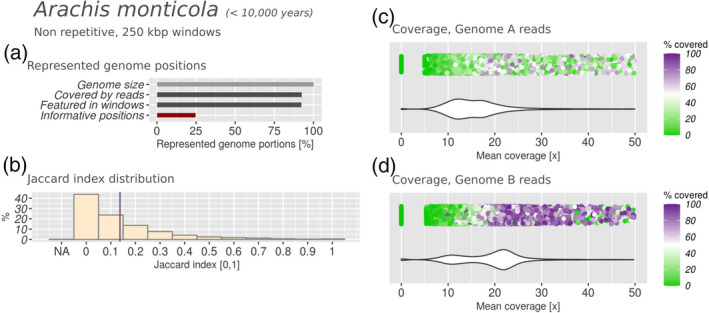
Subgenomic intermixing metrics for wild peanut (*Arachis monticola*). Details as described in Figure 2.

In the genome assemblies of both wild and domesticated tetraploid peanut species, non‐repetitive positions accounted for about 25% of the genome (Figures 6a and 7a); however, these positions were distributed evenly over intervals of 250 Kbp, allowing us to process the mapped reads of the parental species in intervals corresponding to at least 90% of each genome. In domesticated peanut, the bimodal distribution of mean coverage and covered fraction by subgenome B reads (Figure 6d) suggested that subgenome B was highly conserved. The read coverage profile indicated two well‐separated peaks corresponding to 20‐fold and sevenfold coverage, respectively. The latter peak indicated that some reads did not find a well‐conserved match in subgenome B. We attribute these in part to homologous regions in subgenome A, and in part to degraded portions of subgenome B (Figure 6d). In contrast, the two modes in the coverage profile by genome A reads were closer in terms of coverage and density, indicating less sequence conservation in subgenome A (Figure 6c). Despite that, the mean Jaccard index was 0.058, corresponding to fairly non‐intermixed subgenomes, attesting to the fact that subgenome structure conservation does not automatically also imply sequence conservation.

In the wild peanut, the bimodal distributions were less evident and more overlapped in both subgenomes (Figure 7c,d), with the profile from genome A reads being barely bimodal. We observed higher subgenomic intermixing, indicated by a mean Jaccard index of 0.137 (Figures 5b and 6b). Overall, our results showed reduced subgenomic intermixing in domesticated peanut, when compared with its wild relative.

We then turned to the domesticated tetraploid emmer wheat *T. turgidum* ssp. *durum* and its wild relative *T. turgidum* ssp*. dicoccoides* (Priyadarshan, 2019). Both are the result of the hybridization between ancestors of the diploid species *Triticum urartu* (subgenome A) and *Aegilops speltoides* (genome S‐derived subgenome B). Hybridization is considered to have happened multiple times in the history of wheat (Glémin *et al.*, 2019), and has been dated to 0.8–1.5 Mya. Therefore, the two *Triticum* species analysed have an older and more complex genomic history than the peanut, involving approximately 0.8 million years of independent evolution prior to the domestication of *T. turgidum* ssp. *durum*. We mapped genomic sequencing reads from the parental species against the genome assemblies of wild and domesticated emmer wheat and analysed their coverage in intervals of 250 Kbp (Figures S4 and S5). In both cases, the extreme repetitive content of the genome reduced the number of informative non‐repetitive positions to 10–15% of the total genome size; this did not, however, affect the genome fraction represented in intervals of 250 Kbp, which exceeded 95% (Figures S4a and S5a). In both genomes, the mean coverage and covered fraction distributions obtained from subgenome B reads were broad and without a clear peak; in contrast, subgenome A reads resulted in bimodal distributions (Figures S4c,d and S5c,d), suggesting that subgenome A was better conserved in terms of sequence identity with respect to its parental progenitor in both emmer wheat subspecies. The Jaccard index distributions indicated slightly higher intermixing in domesticated emmer wheat (mean 0.083) than in its wild relative (mean 0.059). Hence, the analysis indicated that domesticated emmer wheat subgenomes were slightly more intermixed than those of the wild emmer wheat, although for both species a low mean Jaccard index was found.

Taken together, these results hint at the fact that domestication alone may not be able to explain the extent of subgenomic intermixing observed in domesticated crops. We speculate that a similar analysis performed on a larger number of polyploid crops would reveal more specific trends, shedding light on the interdependency between these two forces.

### Biased fractionation

Hybrid genomes often display plasticity during their evolution. In particular, the balance usually gets tilted to one of the two subgenomes (Edger *et al.*, 2017). This phenomenon is driven by two linked aspects: biased fractionation and subgenome dominance (Cheng *et al.*, 2012). The first refers to the preferential retention, loss or homoeologous conversion of genetic material of a specific subgenome, whereas the latter refers to the preferential expression of genes encoded within one of the subgenomes. In the context of this work, we focused on the first aspect. We asked whether biased fractionation and subgenomic intermixing were correlated or, in other words, whether enhanced genome plasticity (i.e. intermixing) was correlated with preferential allelic conversion or loss of a subgenome.

The effects of biased fractionation can be seen by comparing the sizes of each subgenome with its parental counterpart: a reduction in size is often more prominent in one of the two subgenomes (Bertioli *et al.*, 2019; Bird *et al.*, 2018; Cheng *et al.*, 2012). For each of the species studied we extracted all of the 250‐Kbp intervals where we observed a Jaccard index of ≤0.2 and where either one of the parental species (but not both) covered more than 50% of the informative positions. We consider these regions as unequivocally assigned to one of the two subgenomes. Using the intervals that are uniquely assigned to one parent, we estimated the size of each subgenome in each species. Together with the genome size of the extant relatives of the parental species, we constructed contingency tables (see Experimental procedures) and used Fisher’s combined probability test to evaluate the hypothesis of biased fractionation. We conducted the test under the assumption that both parental genomes collected random mutations at similar rates since the date of hybrid formation and evaluated whether one of the subgenomes had undergone more loss than the other. Another key assumption was that a subgenome size decrease of <1 Mbp would be negligible in the scope of this study but may be statistically significant within the test. Hence, we considered only size changes in the order of Mbp and scaled all genome and subgenome sizes accordingly (Table 1).

**Table 1:** Contingency table used for the biased fractionation analysis, built with subgenomic (S1 or S2) size estimates (Gb), based on this work, and genomic (G) size estimates, derived from published genome assemblies

Scientific name	S1	S2	G1	G2	S1/G1(%)	S2/G2(%)	G1	G2	*P*
*Arachis hypogaea*	0.995	1.221	1.250	1.560	79.60	78.27	*Arachis duranensis*	*Arachis ipaensis*	4.0E–01
*Arachis monticola*	0.572	0.953	1.250	1.560	45.76	61.09	*Arachis duranensis*	*Arachis ipaensis*	**5.8E–16**
*Brassica juncea*	0.276	0.381	0.600	0.591	46.00	64.47	*Brassica oleracea*	*Brassica nigra*	**1.9E–10**
*Brassica napus*	0.206	0.430	0.600	0.485	34.33	88.66	*Brassica oleracea*	*Brassica rapa*	**3.1E–79**
*Chenopodium quinoa*	0.390	0.636	0.452	0.815	86.28	78.04	*Chenopodium pallidicaule*	*Chenopodium suecicum*	**3.2E–04**
*Gossypium* *hirsutum*	1.137	0.788	1.746	0.800	65.12	98.50	*Gossypium* *arboreum*	*Gossypium* *raimondii*	**2.9E–98**
*Nicotiana benthamiana*	0.168	0.104	3.100*	2.400	5.42	4.33	*Nicotiana glauca*	*Nicotiana sylvestris*	6.9E–02
*Nicotiana tabacum*	0.763	1.244	2.400	2.250	31.79	55.29	*Nicotiana sylvestris*	*Nicotiana tomentosiformis*	**3.4E–59**
*Triticum turgidum* ssp. *dicoccoides*	2.632	2.908	4.940	5.700*	53.28	51.02	*Triticum urartu*	*Aegilops speltoides*	**2.1E–02**
*Triticum turgidum* ssp. *durum*	3.717	4.353	4.940	5.700*	75.24	76.37	*Triticum urartu*	*Aegilops speltoides*	1.8E–01

*Values obtained with flow cytometry. *P* values were obtained using Fisher’s test. *P* value set in bold indicate that biased fractionation is observed with statistical significance (*P* < 0.05). Statistical tests were performed based on manticore results with an interval size of 250 Kbp, considering changes of 1 Mbp or more. Parental assignment was performed if *J* ≤ 0.2 and if one of the parents covered >50% of the informative positions exclusively.

We observed statistically significant (*P* < 0.05) biased fractionation in *A. monticola*, *B. juncea*, *B. napus*, *C. quinoa*, *G. hirsutum*, *N. tabacum* and *T. turgidum* ssp. *dicoccoides*, i.e. within all the species tested, with three exceptions. These exceptions were domesticated peanut, domesticated emmer wheat and *N. benthamiana*. Interestingly, no part of the hybrid *N. benthamiana* genome was assigned to a specific parental lineage. In fact, less than half of the total hybrid genome size was covered by reads, and the covered portions were covered by reads from both parents, producing high Jaccard index values. Hence, we could not estimate the portions deriving unequivocally from either parent. We concluded that *N. benthamiana* underwent nearly complete subgenomic deterioration. As for the two peanut species tested, the results differed substantially. Despite both being around 10 000 years old, the subgenomes of the wild peanut were evidently less conserved. Notably, the species displaying the lowest intermixing levels in our study (i.e. quinoa, cotton and smoking tobacco) all showed biased fractionation. Taken together, these results demonstrate the lack of a direct correlation between biased fractionation and subgenomic intermixing.

## DISCUSSION

### Subgenomic intermixing is a multifaceted evolutionary process

In this study we addressed subgenomic intermixing in a wide range of polyploid plant genomes. We mapped parental genomic read data onto each hybrid genome and calculated mean coverage, covered fraction and Jaccard index (*J*) in intervals of 250 Kbp. From the combination of these metrics, we deduced the conservation of the subgenomic sequence and architecture of several hybrid species.

The rates of subgenomic intermixing observed in the 10 species studied ranged from *J* = 0.015 in quinoa to *J* = 0.403 in *N. benthamiana*, averaged over the entire genome. Most species produced a Jaccard index of <0.1, indicating that *N. benthamiana* is exceptional regarding the evolution of its subgenomes. Intermixing levels can differ within the same genus, e.g. wild peanut was twice as intermixed as domesticated peanut (Figures 6b and 7b). They could also differ substantially between species in the same age range, as shown with *N. benthamiana* and quinoa, or being very similar despite the age difference, as showcased by the comparison between quinoa, cotton and smoking tobacco. The comparison of the results obtained from all the species revealed the absence of a direct correlation between hybrid age and the extent of subgenomic intermixing. Overall, a heterogeneity of results was observed, which transcended the phylogenetic proximity as well as the similarity in hybrid age. We speculate that this might depend on the particular selective pressure experienced by different hybrids, through the environment or by the biological properties of a species: metaphase chromosome pairing, transposable elements (Petit *et al.*, 2010), viral defence mechanisms (Bally *et al.*, 2015), the methylation patterns of the parents or the selfing rates.

### Subgenomic intermixing does not correlate with age

The age of the species analysed in this work range from a few thousand years to 5 million years. Interestingly, the analysis of the oldest hybrids in the data set (quinoa and *N. benthamiana*) produced results that are at the extreme opposites of the intermixing spectrum. The subgenomes of a hybrid species diverge from their parental counterparts through the accumulation of random mutations, recombination between homoeologous chromosomes and genome downsizing (Cheng *et al.*, 2018). The effects of these three evolutionary forces accumulate over time, but the rate at which they take place differs because of the different selective pressure and genomic properties of the species. In fact, the results obtained for quinoa (hybridization dated 3.3–6.3 Mya) were similar to those obtained for upland cotton (dated 1.0 Mya) and smoking tobacco (dated 0.4 Mya). *Nicotiana benthamiana*, which is also a tobacco species, is an extremophile plant that is found at sites in the Australian Granites, characterized by a desert‐like environment. Plant vigour is reached early to favour quick reproduction, in a trade‐off with viral defence mechanisms (Bally *et al.*, 2015). Its genome and transcriptome have been sequenced and analysed because of its relevance in biotechnology (Bombarely *et al.*, 2012; Schiavinato *et al.*, 2019). Quinoa, instead, is a versatile food crop adapted to the high‐altitude plains of central South America and is resistant to abiotic stress (Adolf *et al.*, 2012; Jacobsen *et al.*, 2003). *Nicotiana benthamiana* did not undergo a domestication process whereas quinoa was domesticated around 7000 years ago, becoming one of the most relevant food crops of this area (Jarvis *et al.*, 2017). *Nicotiana benthamiana* mainly favours outcrossing (Bally *et al.*, 2018), whereas quinoa has been reported to be mainly self‐pollinating (Risi, 1984). Hence, the two species have grown in different habitats, use different reproduction strategies and were subjected to different sources of selective pressure. Our results showed that these two similarly aged hybrids returned opposite results, with quinoa having its original subgenome structure preserved and with *N. benthamiana* being at an advanced stage of subgenomic intermixing. We also showed that smoking tobacco and upland cotton show subgenomic intermixing rates similar to that of the much older quinoa. Overall, our results showed that older hybrids do not necessarily have highly intermixed subgenomes, despite having had the time to accumulate mutations and undergo genomic rearrangements after hybridization. Hence, we speculate that the slow rate of intermixing observed in these species could be what encouraged the repurposing of genomic material instead of the loss of it, resulting in traits that could be interesting for human agriculture.

### Relationship between domestication and subgenomic intermixing

The results observed with species from the *Arachis* and the *Triticum* genera hinted at a complex relationship between subgenomic intermixing and domestication. Domesticated plants are often spared the selective pressure acting upon their wild relatives, through human intervention. Moreover, most of the selective pressure experienced by domesticated species is related to product traits (e.g. fruit size and quality). Hence, compared with their wild relatives, domesticated species are more likely to retain genomic variation, even in cases where it negatively impacts fitness (Lu *et al.*, 2006), and possibly also rearrangements derived from subgenomic intermixing events.

On the other hand, although less selective pressure is usually experienced by domesticated species, a higher rate of self‐fertilization is also observed as part of what is commonly referred to as ‘domestication syndrome’ (Meyer *et al.*, 2012; Velázquez‐López *et al.*, 2018). For example, the domesticated peanut is remarkably more self‐fertilizing than the wild peanut (Pattee *et al.*, 1998), and we observe lower intermixing in domesticated peanut despite the proximity in time between the formation of the wild progenitor species by hybridization and the domestication event (Bertioli *et al.*, 2019). Self‐fertilization could be expected to reduce subgenomic intermixing. In fact, the genomes contained in the self‐fertilizing gametes originate from the same plant, drastically reducing the impact of parental genetic diversity on the hybrid genome’s stability (Glombik *et al.*, 2020). In the case of wild and domesticated emmer wheat, both are self‐fertilizing plants with outcrossing rates below 5% (Dvorak, 2013; Sahri *et al.*, 2014). We find a higher rate of intermixing in the domesticated emmer wheat. Hence, we can speculate that higher genome plasticity (i.e. subgenomic intermixing) was permitted by a decrease in natural selective pressure following domestication. The relationship between domestication and subgenomic intermixing is therefore highly dependent on the evolutionary context.

In both peanut genomes we show that subgenome B is more conserved, which is also confirmed by its higher mapping rates (Table S2). This agrees with previous findings. In fact, Bertioli *et al.* (2016) proposed that, in terms of long‐range sequence similarity, subgenome A is more diverged than subgenome B in comparison with extant parental relatives. The authors made this conclusion based on an Illumina synthetic long‐read protocol (‘moleculo’) for sequencing the genome of the domesticated hybrid, and by aligning these long reads against the genome assemblies of the diploid parents (Bertioli *et al.*, 2016). With our method we studied the *Arachis* genomes from a different angle, as it is based on short‐read genomic sequencing of the parental progenitors and mapping against the non‐repetitive portions of the derived hybrid genome. These differences aside, our results indicate the same trend. In the domesticated peanut genome it was shown that sequences from subgenome A were detectable in subgenome B much more often than vice versa (14.8 versus 3.1 Mbp) (Bertioli *et al.*, 2019). This indicates that some subgenome intermixing has taken place, and that subgenome A might be more fractionated than subgenome B. In fact, the mean coverage and covered fraction distributions from subgenome A were broader and less sharp than those from subgenome B (Figure 6c), and the biased fractionation results showed a higher fractionation of subgenome A (Table 1). This could suggest a more rapidly evolving subgenome A, which lost more sequence identity to its parental donor compared with subgenome B. Coverage profiles obtained with the wild peanut (Figure 7c,d) underline the same trend. However, the genome of the wild hybrid appears not only more intermixed than its domesticated counterpart, but also less similar to the parental genome donors. All in all, we speculate that the DNA homogeneity produced by self‐fertilization might be what conserved the genome sequence and structure of the domesticated peanut.

The differences observed in wild and domesticated emmer wheat are less striking than those observed in wild and domesticated peanut. We could attribute this mainly to the long time (≥0.8 Mya) that separated the hybridization event from the onset of domestication and the fact that both species are highly self‐fertilizing (Sahri *et al.*, 2014). Hence, domestication traits such as self‐fertilization (Doebley *et al.*, 2006), which are likely to have contributed to the higher conservation of the domesticated peanut genome, do not apply to the domesticated emmer wheat genome. In other words, the genome of domesticated peanut is more conserved than the genome of its wild relative, both in terms of intermixing rate and similarity to the parental subgenome donors. On the other hand, the genome of domesticated emmer wheat is only more conserved than the genome of its wild relative in terms of similarity to the parental subgenome donors. Following this observation, the combination of time, domestication traits, and genomic and environmental selective pressures should be considered when studying the interaction of subgenomes in a hybrid species.

Overall, our results suggest that the rate of outcrossing might play a role in enhancing subgenomic intermixing. The two most outcrossing species in our data set (*N. benthamiana* and the wild peanut) showed the highest intermixing rates. Outcrossing species might also be more prone to interspecific crossing, which would further accentuate subgenomic intermixing through introgression events. Hence, we concluded that domestication traits such as self‐fertilization cannot explain the subgenomic intermixing state of a hybrid species on their own, but they may if the hybridization and the domestication events are both recent and occurred roughly at the same time.

### Intermixing is independent from subgenome sequence conservation

The subgenomes of a hybrid may evolve through intermixing, the accumulation of variation and biased fractionation. According to our observations these seem to be relatively independent dynamics. In fact, two subgenomes may be intermixed but also retain sequence identity to their parental counterparts if blocks of DNA sequences are shuffled between homeologs without accumulating variation. On the other hand, two subgenomes may not undergo much intermixing but accumulate variation to an extent that their sequence does not resemble the parental sequence anymore. The divergence of the subgenome sequences from their parental counterparts is reflected directly in the mapping rates of the short‐read data sets used to separate the assemblies (Table S2). These could differ even within the same genus, as shown with the species from the *Brassica* genus: 70–72% of parental donor reads from subgenome A mapped to Chinese mustard (AB hybrid), whereas 87–90% mapped to rapeseed (AC hybrid). Two subgenomes may also follow different evolutionary routes within the same genome, as showcased by many of the species tested, e.g. quinoa, smoking tobacco and domesticated emmer wheat (Figures 4, S2 and S5). This was particularly clear when looking at the biased fractionation results, where most of the species, independently from the measured intermixing levels, showed statistically significant biased fractionation. Hence, we stress that subgenomic intermixing should be considered as an independent evolutionary phenomenon, which may or may not align with the trends observed with other evolutionary forces, such as biased fractionation and variation.

Our results also suggested that subgenome size and biased fractionation are not correlated. If fractionation happened randomly, that is, targeting both subgenomes with the same intensity, one could argue that the larger subgenome of a hybrid may be subjected to more fractionation because of the larger size. Our results show that this is not a valid assumption. For example, in rapeseed and in Chinese mustard the smaller parental subgenome lost more genetic material (Table 1). In both species, assignment to subgenome A with sequencing reads was less successful than assignment to the other parent, i.e. subgenome C in rapeseed and subgenome B in Chinese mustard. In rapeseed, Chalhoub *et al.* (2014) reported a higher rate of allele migration of subgenome C to subgenome A than vice versa, which explains the biased fractionation that we observe: the retention of subgenome C is favoured. Given our results we can also suspect that, in Chinese mustard, homoeologous conversion also happens more often from subgenome B to subgenome A. On the contrary, in upland cotton, quinoa and smoking tobacco the subgenomes that underwent more loss are the larger ones among the two parental genomes. In our upland cotton results the portion assigned to the larger subgenome A with genome A reads was drastically lower than that assigned to the smaller subgenome D with genome D reads: 65 versus 98%, respectively (Table 1). This correlates with previous findings, where it has been shown that the subgenome A of upland cotton was subject to much more intense allele conversion to subgenome D than vice versa (Guo *et al.*, 2014). The quinoa and the smoking tobacco results showed similar trends, with their larger subgenomes showing more fractionation.

### Consequences of wrong parental subgenome donor choice

The choice of a potential parental progenitor genome used as a source for mapping sequencing reads against the genome of the hybrid may affect the ability to distinguish subgenomes. As a proof of concept, we demonstrated that using sequencing reads from other sources than the parental genome donors of rapeseed generated unimodal coverage profiles (Figure 3). In fact, when using data from the closely related but non‐parental genome donor species *B. nigra* (Figure 3a), the covered fraction did not surpass 40% of the interval length. To ensure that *B. nigra* genomic reads did not have any intrinsic quality issue, we also mapped them onto the Chinese mustard genome (AB hybrid) (Figure S1). Reads covered 45–50% of the genome positions, distributed within 80–85% of the genome. The importance of the correct progenitor choice is also found in the context of the two quinoa subgenome B parental candidates, *C. ficifolium* and *C. suecicum*. In this work we showed results obtained with genomic reads from *C. suecicum*. Nevertheless, we also analysed *C. ficifolium* reads. Although *C. ficifolium* sequencing reads have a lower mapping rate than those of *C. suecicum*, the separation of quinoa into subgenome B and non‐subgenome B intervals is more pronounced with *C. ficifolium*. This result highlights that even slight differences between candidate parental species may result in different coverage and subgenomic intermixing profiles.

Overall, we conclude that analyses using wrongly chosen parental progenitor candidates are expected to result in less clearly separated bimodal coverage distributions. We must, however, be aware that analysing the correct parent may also produce a unimodal distribution if enough variation has accumulated, as exemplified by *N. benthamiana* and emmer wheat (Figures 5, S4 and S5). Therefore, the presence of a bimodal coverage profile can serve as solid evidence that the chosen species is indeed the extant relative of the parental subgenomic donor, or a very closely related species. However, unimodal coverage profiles do not necessarily indicate a wrong choice of parental subgenomic donor. As a screening method, we suggest that the analysis approach introduced in this work and implemented in the manticore pipeline may facilitate decisions when several subgenome progenitor candidates are under assessment.

## Experimental procedures

### Species selection and identification of unique portions in genome assemblies

Allopolyploid species with publicly available genome assembly, genomic reads of the subgenome donors, and a known hybridization date were selected (Table S3). If present, we also downloaded information on annotated repetitive elements in the genome assemblies. In cases when no repeat annotation was available, *de novo* repeat identification was performed. Catalogues of known repetitive elements were used as a template to model repeats on a draft assembly. Alternatively, such a catalogue was generated with repeatmodeler (‐engine ncbi) (Smit and Hubley, 2008). The catalogue was then passed on to repeatmasker (‐engine ncbi ‐‐gff ‐‐lib library.fa) (Smit *et al.*, 2013). The coordinates of the repetitive elements obtained from repeatmasker were converted to a non‐redundant browser extensible data (BED) file using bash shell commands and bedtools merge (Quinlan and Hall, 2010). The merged BED file was reversed using bedtools complement to obtain the coordinates of non‐repetitive regions. Stretches of ‘N’ (gaps) in assemblies were excluded from these positions.

### Sequencing read processing and downsampling

Illumina paired‐end read data (Table S3) were trimmed with trimmomatic 0.35 (Bolger *et al.*, 2014) using the parameters PE ILLUMINACLIP:TruSeq3‐PE.fa:2:30:10 LEADING:3 TRAILING:3 SLIDINGWINDOW:4:25 MINLEN:50. The trimmed reads were down‐sampled with the custom python script ‘downsample‐reads.py’ provided with this work as part of our pipeline (manticore, see below) to a sequencing depth between 10‐ and 15‐fold, based on the size of the corresponding hybrid genome. Because all nucleotides have a similar chance of getting sampled (Lander and Waterman, 1988) we consider this coverage to be sufficient to ensure that our experimental set‐up takes into consideration the whole genome. The expected coverage only represents an estimate for the down‐sampled libraries, as mapping was conducted on the hybrid genomes but the reads were generated from the parental genomes. In the ideal case of two subgenomes being of equal size, a parental read library will produce a coverage over its corresponding subgenome that is twice the coverage estimated using the total hybrid genome size. Hence, a library containing 15‐fold of the hybrid genome size will produce a 30‐fold coverage over its corresponding subgenome, provided that this is half the size of the total hybrid genome. An entirely conserved subgenome is not expected, however, because of sequence variation and the loss of genetic material. Moreover, the two subgenomes (and their parental counterparts) often differ in size and before read mapping these sizes are usually unknown. These factors make the expected coverage fluctuate around 30‐fold. Hence, we expected read coverage distributions to have a peak between 15‐ and 45‐fold. A secondary peak at a lower coverage is also expected, as reads from one parental donor might also find matching regions in the other subgenome.

### Read data analysis

A pipeline named manticore was developed to perform all the operations required in our analysis; the main steps are represented in Figure 1 and comprise the mapping of parental short‐read data on a hybrid genome, filtering steps and coverage analysis. The pipeline executes a series of python and r scripts through a single command from the command line, using parameters that can be adapted by the user according to the input data. Adjustable parameters comprise, for example, the size of the intervals considered (here 250 Kbp), minimum coverage (here 5×), number of threads, and maximum memory usage; a complete usage manual is provided on the GitHub page.

The following steps are all performed within the Manticore pipeline and were applied as such in the analyses of this work. The parental sequencing reads are mapped onto the hybrid genome assemblies using HISAT2 (Kim *et al.*, 2015) (v2.1.0). Unless mapping parameters are specified by the user, the HISAT2 default parameters are used (here: ‐k 5 ‐‐score‐min L,0.0,‐0.6 ‐‐mp 6,2 ‐‐rdg 5,3 ‐‐rfg 5,3 ‐‐no‐softclip ‐‐no‐spliced‐alignment). The mapping results are then filtered and sorted using the python module Pysam built around samtools (Li *et al.*, 2009), filtering out secondary alignments (‐F 0x0100) and unmapped reads (‐F 0x4). The sorting is conducted according to genome coordinates. Coverage per position is calculated with Pysam using the filtered reads. Only positions with a coverage value greater or equal to the one specified via the input parameters are retained (here: minimum 5× coverage).

Only non‐repetitive positions were considered in this analysis. This is controlled by the user with a parameter, passing a BED file containing positional ranges to be included. If the user passes such a file, only positions within the intervals are retained. In our analysis, a BED file for each species was prepared containing the non‐repetitive positions in their genome assemblies and which was passed to the pipeline.

The coverage per position is analysed in intervals of adjustable size (here: 250 Kbp). The analysis of these intervals represents the core set of functions of the pipeline. The interval size of 250 Kbp was chosen after attempting the analysis with intervals of 50 and 500 Kbp. A candidate intermixed interval is detected when both parental progenitors produce coverage inside the interval at an expected mean coverage (details below). The interval size of 250 Kbp was chosen because larger interval sizes (500 Kbp) resulted in a larger number of candidate intermixed intervals, whereas smaller interval sizes (50 Kbp) failed to detect intermixing in most cases.

For each interval, several metrics are computed from the pipeline: (i) interval mean coverages, derived from each parental read data set independently; (ii) interval covered fractions, derived from each parental read data set; and (iii) interval Jaccard index, computed using the parental coverage values detected within the interval. Intervals are considered to carry sufficient information to study intermixing only if they contain at least 1000 non‐repetitive positions where reads can map, at least 1000 of the total positions available are covered and the covered positions amount to at least 10% of the total positions available.

A limit of the use of read mapping for this purpose is the ability to differentiate between truly intermixed regions and regions of high homology. In other words, to distinguish intermixed intervals from those where subgenome donors carry nearly identical sequences and consequently align on the same positions in the hybrid genome. This issue is tackled in the pipeline by dividing each interval into bins (here, 10 bins of 25 Kbp each). For each bin, the mean coverage and the covered fraction by each parental read library are computed separately, and then used to compute a Jaccard index for the whole interval between parental read coverages. First, the presence or absence of coverage by each parental read set is assessed in each bin. The results are formalized in two True/False (i.e. presence/absence) Boolean vectors, one for each parental read library. The length of the vector corresponds to the number of bins (here, 10 bins).

A series of filtering and normalization steps are applied to each bin in an interval prior to the Jaccard index calculation. First, if a parental read data set covers less than 10% of the non‐repetitive positions of a bin, the coverage contribution in that bin is considered insufficient and the bin is marked as ‘False’ for that parent. Second, a coverage normalization is applied. In fact, due to the phylogenetic proximity of the parents of the hybrid species, many bins are covered by both parental read data sets as a result of high sequence identity. To tackle this, a mean coverage from each parental read data set in each bin is computed. Despite the genome sequence identity between parental progenitors, one of the two will likely be closer to the hybrid’s genome sequence represented in the bin. This, in turn, will result in fewer mismatches or gaps in the mapping and will produce a higher mean coverage per position within the bin.

manticore normalizes the coverages obtained with parental read mapping by the observed sequencing depth of the read data set on the hybrid genome (omitting uncovered positions). In detail, manticore computes a ratio between the observed mean coverage per position over the whole genome produced by the first‐declared sequencing read data set and that obtained from the second (the declaration order depends on the command‐line parameters). This factor is then applied to the mean coverages produced with the second declared read data set, computed in each bin. Following this normalization, in bins where the two mean coverages are not within ±25% of the coverage of the other parent, the lower mean coverage is scored as ‘False’. This range ensures that fluctuating coverage would not have a huge impact on the assignment of the presence/absence status. As a result of the normalization, bins are more likely to be labelled ‘True’ for only one parent, i.e. one primary covering parent gets identified per bin based on the differences in parental coverages. The bins that are still considered as covered by both parents after normalization are those that point to intermixing.

Finally, a Jaccard index is computed dividing the number of bins with coverage contribution from both parents by the number of bins with coverage contribution from at least one parent. In mathematical terms, the numerator is the intersection of the ‘presence’ bins (both parents are marked as ‘True’) and the denominator is the union (at least one parent is marked as ‘True’). The Jaccard index ranges from 0 to 1. A value close to 0 indicates low intermixing because few bins showed a contribution from both parental read data sets. A value close to 1 indicates high intermixing because both parental read data sets produced coverage in the majority of the bins. The output of the pipeline is organized by interval (250 Kbp) and comprises the mean coverages by each parental read data set, the covered fractions by each parental read data set and the Jaccard index. Each threshold declared in this paragraph can be adjusted by the user with a command‐line parameter.

### Parental assignment of genes based on sequence homology

*Brassica napus* transcript sequences were downloaded from http://www.genoscope.cns.fr/brassicanapus and were aligned against transcript sequences from *B. oleracea* and *B. rapa* (i.e. the known parental species that have hybridized during the formation of *B. napus*), obtained from Ensembl (http://plants.ensembl.org/Brassica_rapa, http://plants.ensembl.org/Brassica_oleracea). The alignment was conducted with blastn (Camacho *et al.*, 2009). The hit with the highest identity (min. 70%), the lowest e‐value (max 0.01) and the highest score was selected for each transcript and used to assign it to the corresponding parental progenitor. Criteria were considered in this order of priority: e‐value (lowest), percentage identity (highest) and score (highest). In the case where two alignments produced the same e‐value, the alignment with the highest identity was chosen, and in the case where the identity was also equal, the alignment with the best score was chosen. Transcripts with multiple alignments of the same quality to transcripts of both parental species were left unassigned.

### Parental assignment of genes based on interval assignment

Genomic intervals with a Jaccard index of ≤0.5 and a covered fraction of >50% from a single parental progenitor were selected as computed by our pipeline manticore. The intervals were assigned to the parental progenitor providing the covered fraction of >50%. The genes contained in such intervals were assigned to the same parental progenitor to which the interval was assigned. The assignment was performed twice, with interval sizes of 250 and 25 Kbp, respectively.

### Fisher’s test to evaluate biased fractionation

The manticore pipeline also computes the portion of a hybrid genome that can be unambiguously assigned to either parent. The proportion of the assigned genomic regions provides information about the individual size of the subgenomes, and thus enables us to compare the size of the subgenome with the size of the parental donor genome, showing whether both subgenomes retain similar sizes or whether one subgenome was preferentially lost (i.e. biased subgenome fractionation). For manticore to consider an interval as ‘unambiguously assigned’, the following criteria must be met: a Jaccard index of ≤0.2 and >50% of positions covered by a single parental sequencing read data set. The maximum Jaccard index value can be controlled with a parameter. These two criteria are evaluated on the positions selected in the coverage per position calculation.

If a BED file is specified (see above), only the positions contained within this file will be used in the calculations. However, if the criteria are met, manticore assigns the entire interval (here, 250 Kbp) to the specific parent. A contingency table to evaluate the relationship between subgenomic size and parental donor genomic size was constructed using: (i) the total unambiguously assigned positions, as assessed by manticore; and (ii) the genome size of the parental species, retrieved from the literature. Each line in the contingency table featured the size of subgenome 1 (S1), the size of subgenome 2 (S2) and the difference in size between a parental genome and the corresponding subgenome (G1 – S1 and G2 – S2). A Fisher’s test was performed on the contingency table, to evaluate whether the reduction in size of one subgenome was significantly larger than the reduction in size of the other subgenome within each hybrid. A statistically significant result (*P* < 0.05) indicated biased fractionation. Subgenome size changes below 1 Mbp were disregarded. Consequently, each size was expressed in Mbp, to avoid any overestimation of significance levels (i.e. size changes of <1 Mbp). The Fisher’s test for the contingency table was performed with an r script.

### Hardware and scripting

The manticore pipeline was coded in python 3.6. The architecture of the python code is constructed upon the modules biopython 1.73, pysam 0.15.3, numpy 1.17.3, pandas 1.0.3 and dask 2.12.0. The interpreter for r scripts (rscript) and the r package ggplot2 3.2.1 are used for plotting. Gene set comparisons were performed using perl 5. The manticore pipeline was run on the Vienna Scientific Cluster 3 (VSC3), a high‐performance computing cluster operating on Scientific Linux 6.6. Each species was analysed with comparable resources, that is, 32 cores and 100 Gb random‐access memory (RAM), with a computational time of approximately 2 h per genome.

## Supporting information


**Figure S1**. Subgenomic intermixing metrics for Chinese mustard (*Brassica juncea*).

**Figure S2**. Subgenomic intermixing metrics for smoking tobacco (*Nicotiana tabacum*).

**Figure S3**. Subgenomic intermixing metrics for upland cotton (*Gossypium hirsutum*).

**Figure S4**. Subgenomic intermixing metrics for wild emmer wheat (*Triticum turgidum* ssp. *dicoccoides*).

**Figure S5**. Subgenomic intermixing metrics for domesticated emmer wheat (*Triticum turgidum* ssp. *durum*).

**Figure S6**. Subgenomic intermixing metrics for native Australian tobacco (*Nicotiana benthamiana*) within coding sequences (CDS).

**Table S1.** Genome size, ploidy, estimated hybridization date and candidate parents of each hybrid genome used in this study, and genome size and ploidy of each candidate parental progenitor of the hybrid species.

**Table S2.** Mapping rates obtained from manticore for each hybrid species analysed using genomic reads from its candidate parents.

**Table S3.** Sources of data used in this study.

## Data Availability

The repeat annotations for *B. juncea*, *N. benthamiana* and *N. tabacum* are available at http://bioinformatics.boku.ac.at. All other data used in this study were obtained from public sources (Table S3). The Manticore pipeline can be downloaded from the public GitHub repository: https://github.com/MatteoSchiavinato/manticore.git.
