# Supplementary figures and images for: Subgenome evolution in allotetraploid plants
Source: Plant J. 2021 Mar 24;106(3):672–88. doi: 10.1111/tpj.15190 (PMC8251528; doi:10.1111/tpj.15190)

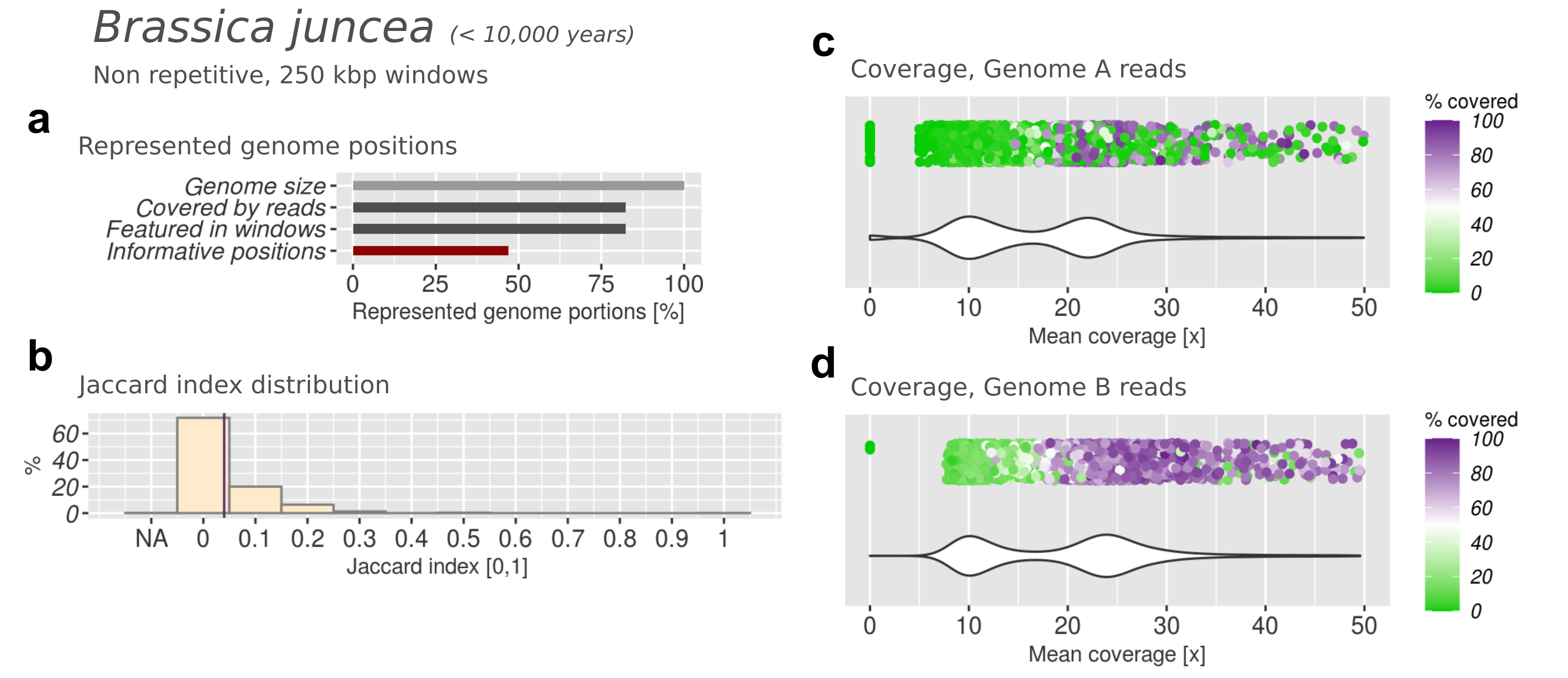

Supplement: Supplementary file 1 — Figure S1. Subgenomic intermixing metrics for Chinese mustard (Brassica juncea). [file TPJ-106-672-s001.png]

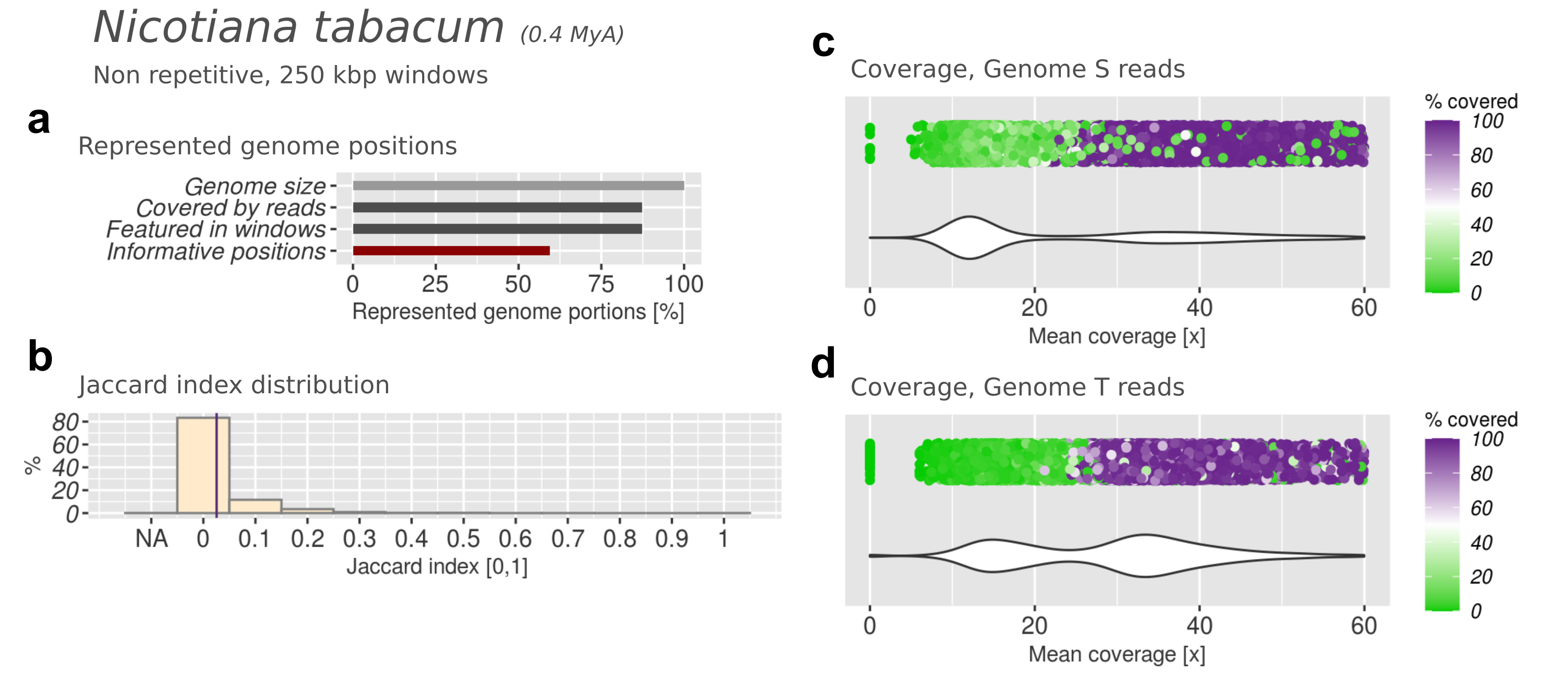

Supplement: Supplementary file 2 — Figure S2. Subgenomic intermixing metrics for smoking tobacco (Nicotiana tabacum). [file TPJ-106-672-s004.png]

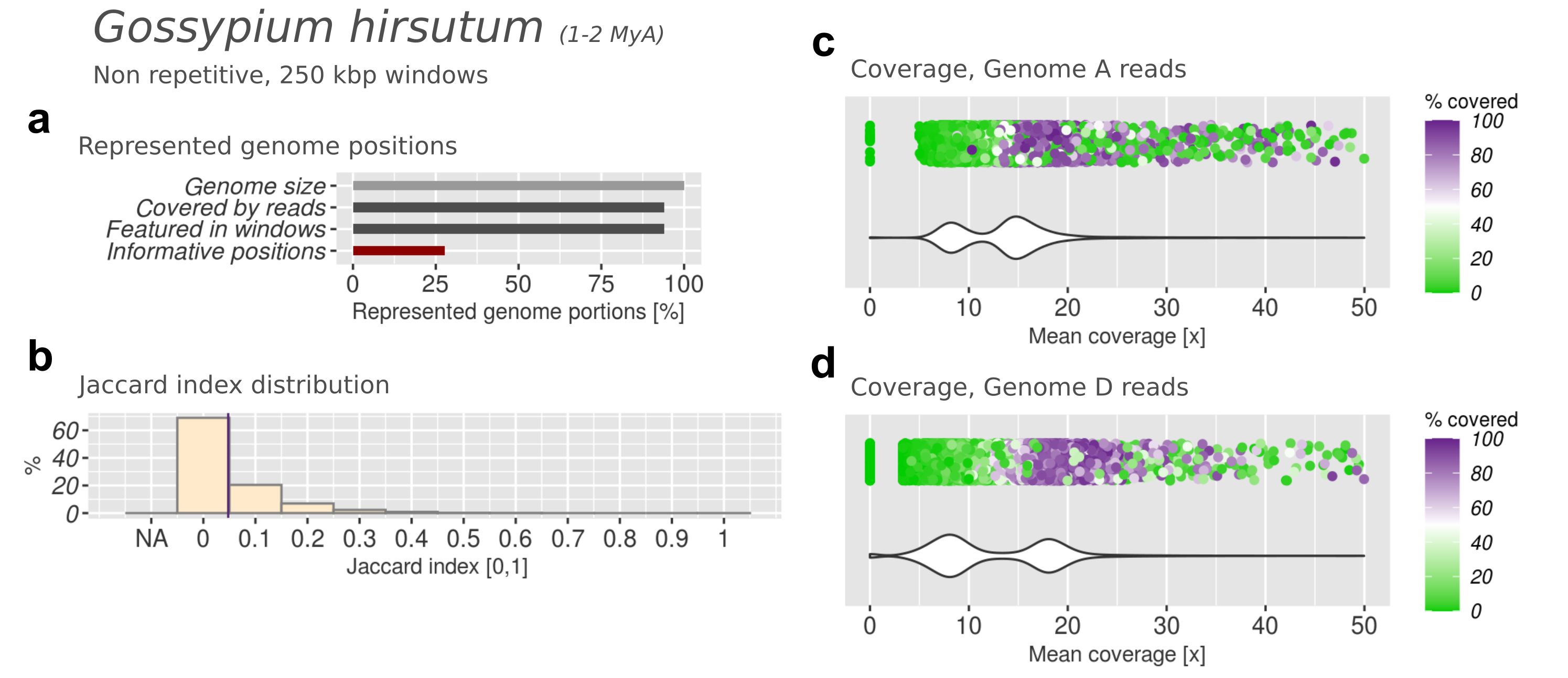

Supplement: Supplementary file 3 — Figure S3. Subgenomic intermixing metrics for upland cotton (Gossypium hirsutum). [file TPJ-106-672-s003.png]

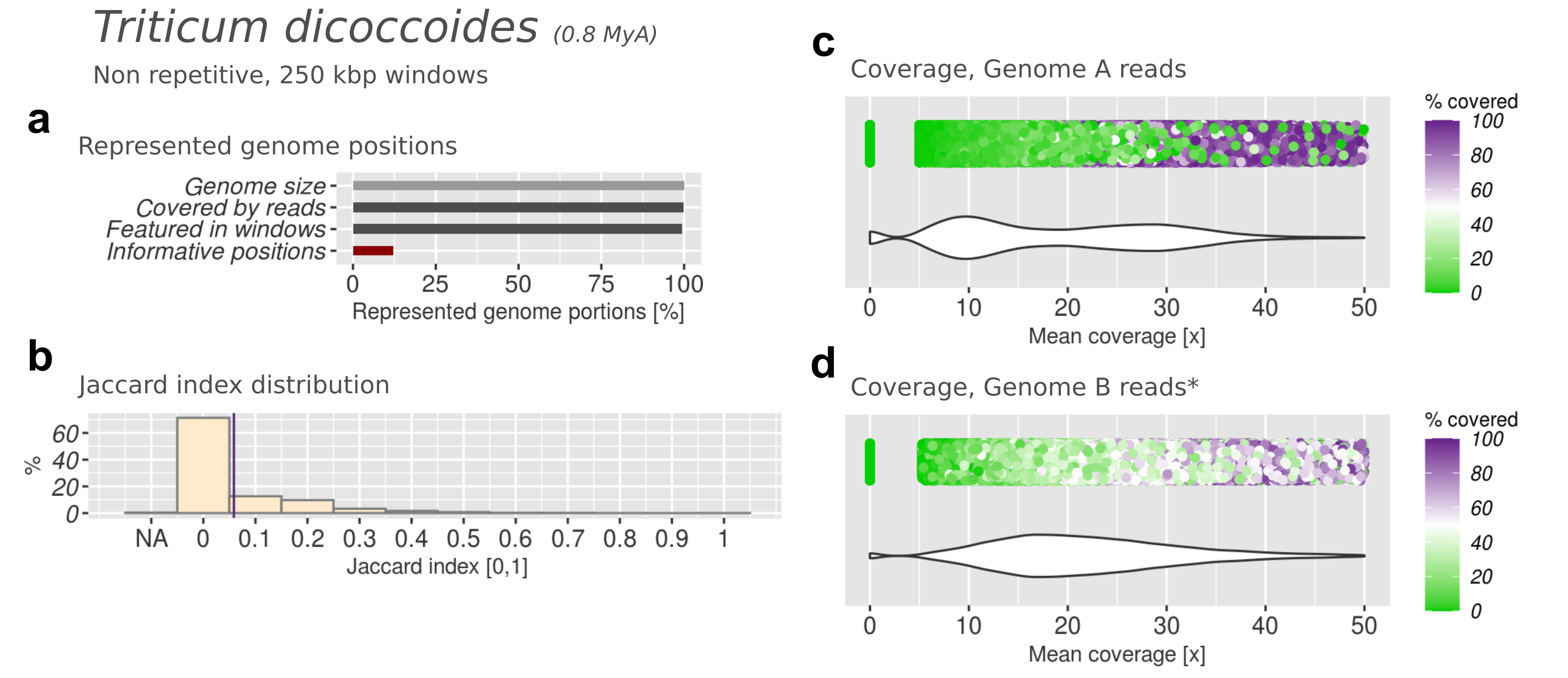

Supplement: Supplementary file 4 — Figure S4. Subgenomic intermixing metrics for wild emmer wheat (Triticum turgidum ssp. dicoccoides). [file TPJ-106-672-s008.png]

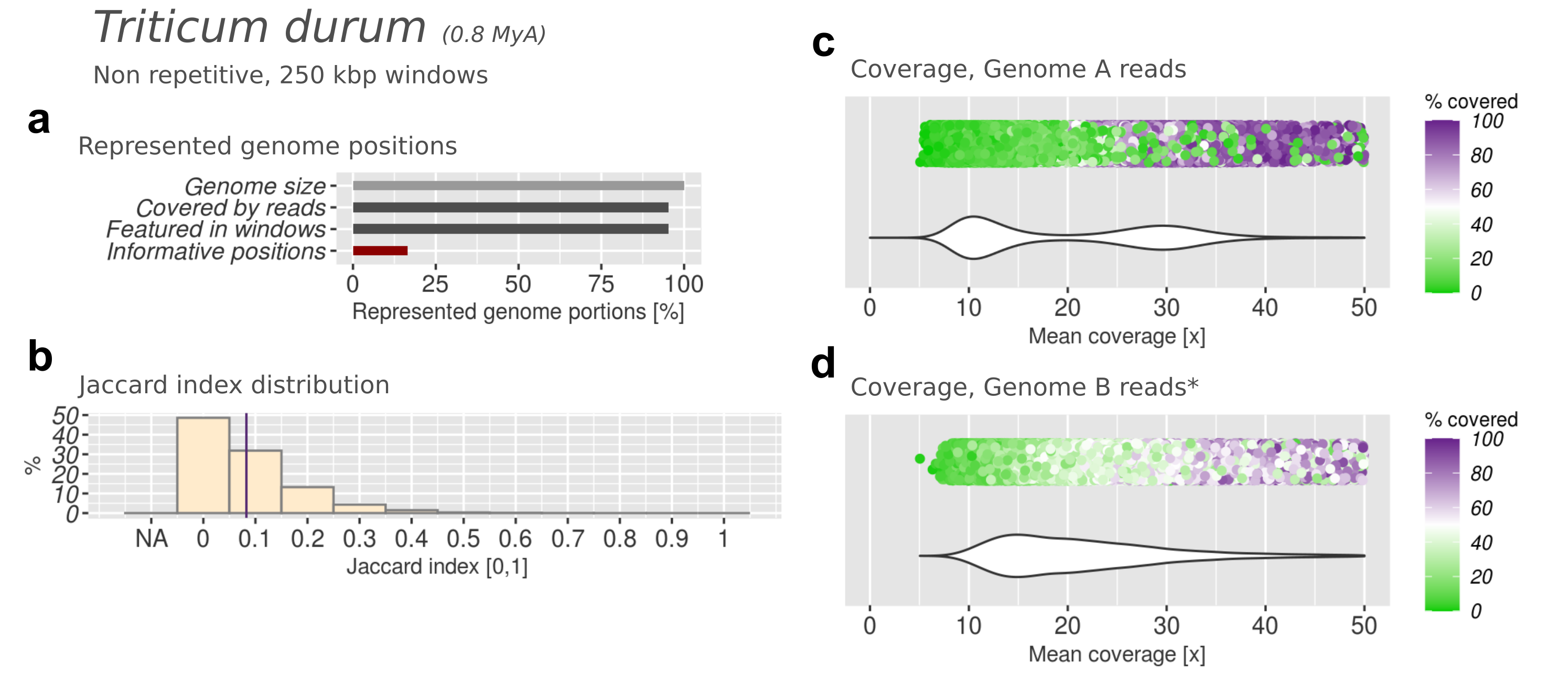

Supplement: Supplementary file 5 — Figure S5. Subgenomic intermixing metrics for domesticated emmer wheat (Triticum turgidum ssp. durum). [file TPJ-106-672-s005.png]

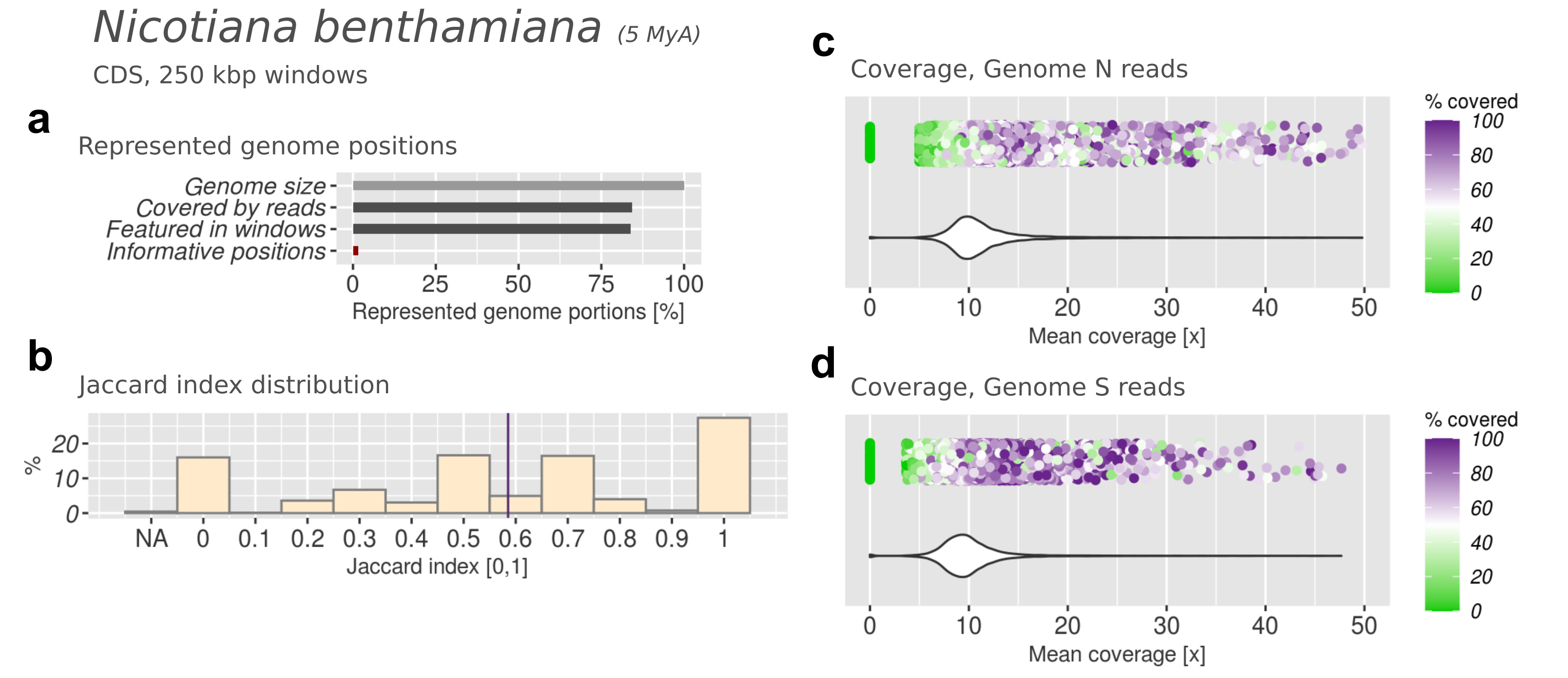

Supplement: Supplementary file 6 — Figure S6. Subgenomic intermixing metrics for native Australian tobacco (Nicotiana benthamiana) within coding sequences (CDS). [file TPJ-106-672-s006.png]
